# Review on Design, Characterization, and Prediction of Performance for Asphalt Materials and Asphalt Pavement Using Multi-Scale Numerical Simulation

**DOI:** 10.3390/ma17040778

**Published:** 2024-02-06

**Authors:** Wentao Wang, Linbing Wang

**Affiliations:** 1 National Center for Materials Service Safety, University of Science and Technology Beijing, Beijing 100083, China; wentaowang@ustb.edu.cn; 2 The Sensing and Perception Lab, School of Environmental, Civil, Agricultural and Mechanical Engineering, University of Georgia, Athens, GA 30602, USA

**Keywords:** asphalt materials, asphalt pavement, material property design, pavement performance prediction, multi-scale numerical simulation

## Abstract

Asphalt pavement, which is mainly made up of the asphalt mixture, exhibits complicated mechanical behaviors under the combined effects of moving vehicle loads and external service environments. Multi-scale numerical simulation can well characterize behaviors of asphalt materials and asphalt pavement, and the essential research progress is systematically summarized from an entire view. This paper reviews extensive research works concerning aspects of the design, characterization, and prediction of performance for asphalt materials and asphalt pavement based on multi-scale numerical simulation. Firstly, full-scale performance modeling on asphalt pavement is discussed from aspects of structural dynamic response, structural and material evaluation, and wheel–pavement interaction. The correlation between asphalt material properties and pavement performance is also analyzed, and so is the hydroplaning phenomenon. Macro- and mesoscale simulations on the mechanical property characterization of the asphalt mixture and its components are then investigated, while virtual proportion design for the asphalt mixture is introduced. Features of two-dimensional and three-dimensional microscale modeling on the asphalt mixture are summarized, followed by molecular dynamics simulation on asphalt binders, aggregates, and their interface, while nanoscale behavior modeling on asphalt binders is presented. Finally, aspects that need more attention concerning this study’s topic are discussed, and several suggestions for future investigations are also presented.

## 1. Introduction

Asphalt pavement exhibits complicated mechanical behaviors under the combined effects of moving vehicle loads and external service environments. For the asphalt mixture, which is one of the main elements that construct asphalt pavement layers, its comprehensive properties directly determine the service quality of the entire road. Accordingly, it is essential to adequately characterize the performance of asphalt materials and asphalt pavement from full scale to nanoscale, followed by material design and performance prediction, both of which together guarantee the service life of asphalt pavement. Multi-scale methods of testing methods and numerical simulation are often applied to evaluate both material properties and structural performance of asphalt pavement. The length ranges of the research objective’s feature size for the macroscale, mesoscale, microscale, and nanoscale include “larger than 10^−1^ m”, “10^−5^~10^−1^ m”, “10^−8^~10^−5^ m”, and “smaller than 10^−8^ m”, respectively. The research scale for asphalt pavement structure is the full scale, which also belongs to the macroscale. For numerical methods, several commercial software, such as ABAQUS of version 6.14 [1], ANSYS of version 19.0 [2], COMSOL of version 6.2 [3], PFC of version 6.0 [4], and Materials Studio of version 2023 [5], are usually adopted to, respectively, conduct finite element method (FEM) modeling, discrete element method (DEM) modeling, and molecular dynamics (MD) simulation for the asphalt pavement and the asphalt mixture.

Compared with performance characterization using experimental methods, numerical simulation can help to obtain abundant behavior features for asphalt materials and asphalt pavement combined with a few limited numbers of trials, especially suitable for research studies concerning several experiments with high costs, such as the full-scale accelerated pavement testing (APT) method [6] and the X-ray computed tomography (CT) scanning method [7]. Moreover, some important service performances, e.g., seepage flow [8] and particle interlocking behaviors inside the asphalt mixture [9] and wheel–pavement interaction with a high driving speed [10], are more effective to be explored by numerical simulation rather than experimental methods. In this case, it is meaningful to firstly review how numerical simulation can help to characterize, design, and predict the performance of asphalt materials and asphalt pavement, as well as explore the correlation between material properties and structural behaviors.

Several research studies have reviewed performance characterization for asphalt materials and asphalt pavement. Fang et al. [11] reviewed aggregate gradation from aspects of theory and design methods and summarized its impact on pavement performance. Schuck et al. [12] reviewed the spatial distribution of air voids inside the asphalt mixture. Plessis and Boshoff [13] summarized the application of CT technology on the asphalt mixture. Wang et al. [14] and Chen et al. [15] reviewed the performance characterization of asphalt materials using the MD method. Tan et al. [16] reviewed the research progress of asphalt binder’s microstructures and components, while Han et al. [17] summarized the effect of rejuvenators on asphalt binders concerning microscopic experimental and numerical methods. It can be found that these studies mainly focus on several branches of research concerning the properties of the asphalt mixture. However, it is essential to comprehensively review and summarize the research progress of design, property characterization, and performance prediction for asphalt materials and asphalt pavement concerning multi-scale numerical simulation from an entire view. Moreover, it is also necessary to make efforts to analyze the correlation between material properties and structural performance, which can lay vital foundations for their performance prediction.

**Figure 1:**
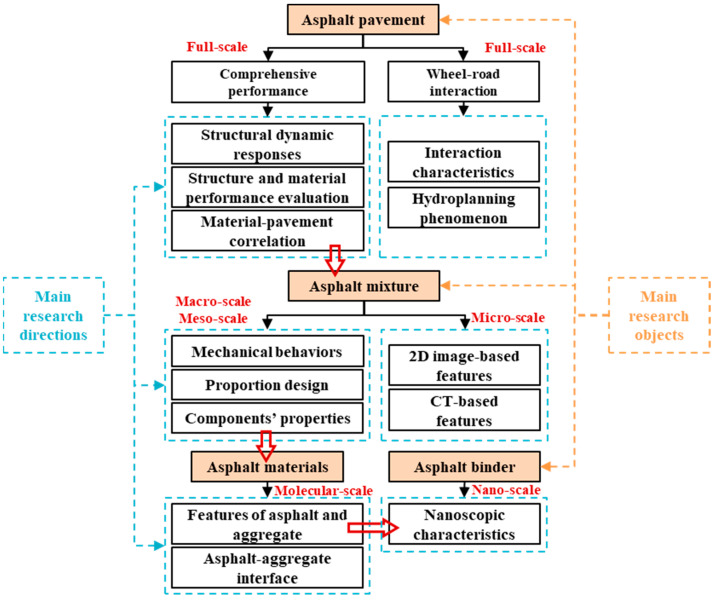
Article framework.

The objective of this study is to review the research concerning aspects of design, characterization, and prediction of performance for asphalt materials and asphalt pavement based on multi-scale numerical simulation. Relevant studies were divided into several main research directions at multiple scales based on different research objects, such as asphalt pavement, the asphalt mixture, raw asphalt materials, and asphalt binders. The framework of this study is shown in Figure 1. Firstly, full-scale numerical simulation on the performance of asphalt pavement is analyzed from aspects of structural dynamic response, structural and material evaluation, and wheel–pavement interaction. The correlation between asphalt material properties and pavement performance is discussed, and so is the hydroplaning phenomenon. Macro- and mesoscale simulations on mechanical property characterization of the asphalt mixture and its components are then investigated, while virtual proportion design for the asphalt mixture is also introduced. Features of two-dimensional (2D) and three-dimensional (3D) microscale modeling on the asphalt mixture are summarized, followed by MD simulation on the asphalt binder, aggregate, and their interface, while nanoscale behavior modeling on the asphalt binder is presented. Finally, aspects that need more attention concerning this study’s topic are discussed, and several suggestions for future investigations are also presented.

## 2. Full-Scale Performance Modeling on Asphalt Pavement

### 2.1. Comprehensive Performance of Asphalt Pavement

#### 2.1.1. Structural Dynamic Response Analysis

Numerical simulation makes it possible to investigate the characteristics of dynamic responses of asphalt pavement under vehicle loading in simple or multiple service environments [18,19,20,21,22], which can help to present the health status of asphalt pavement structure and to further predict the residual service life [23,24,25,26]. Sun et al. [3] constructed a 3D FEM model for the asphalt pavement under multi-field coupling, which included hydromechanical coupling and thermal hydromechanical coupling, and the dynamic response characteristics of saturated structure were numerically analyzed. Yang et al. [27] built a 2D DEM model for asphalt pavement with multilayer subgrade and road surface to analyze its microscopic state mechanics response under a moving vehicle load, while relevant material parameter values for different layers were obtained by conducting mechanical tests with relevant DEM models.

**Figure 2:**
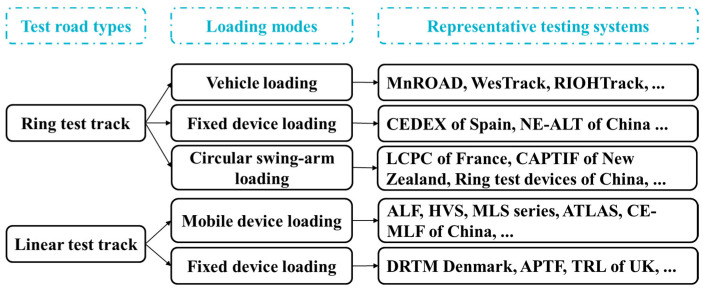
Typical APT systems.

**Figure 3:**
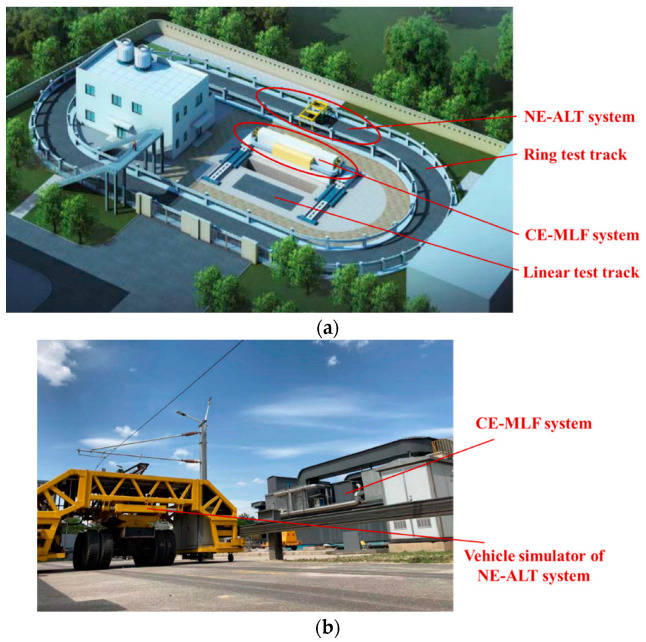
The full-scale APT systems at the USTB, China: (**a**) layout of the entire APT system; (**b**) the full-scale CE-MLF and NE-ALT systems.

Field tests are often conducted using full-scale accelerated pavement testing (APT) systems to validate the effectiveness of the analyzed results of numerical simulation and modify relevant numerical models for better performance prediction of asphalt pavement. The APT systems can be directly divided into two typical categories according to the test road types, which are ring test track and linear test track. Taking the forms of the applied vehicle loads into account, these APT systems can be further classified into the loading modes of real trucks, fixed devices, circular swing arms, and mobile devices [28,29]. Typical APT systems are presented in Figure 2. In particular, as shown in Figure 3, the APT systems at the University of Science and Technology Beijing combine both ring and linear test tracks together to present comprehensive main functions of existing APT systems [30], which are the Natural Environment–Automatically Loaded Track (NE-ALT) system and the Controlled Environment–Multiaxial Loading Facility (CE-MLF) system. In the NE-ALT system, the vehicle simulator moves on the ring test track with the desired speed and load in a precisely controlled path, while the service environment parameters can be strictly controlled for the linear test track in the CE-MLF system, such as temperature, rainfall, ultraviolet radiation, and groundwater table. The purpose of conducting full-scale accelerated loading tests is to obtain mechanical responses of testing roads on the basis of simulating service environment and vehicle load conditions as real as possible, which can lay a solid trial foundation of validation for numerical simulation.

Wang et al. [31] compared the dynamic responses of asphalt pavement in dry and saturated conditions under moving vehicle loads using 2D FEM models and then conducted full-scale APT tests in the same loading parameter combination, which were 20 km/h vehicle speed and 0.7 MPa vehicle wheel grounding pressure, to validate the numerical analyzed results [32]. Peng et al. [33] constructed the three-dimensional 3D DEM model of asphalt surface layers to evaluate its mechanical responses under moving traffic loads, and the numerical results were validated by field tests using a self-developed full-scale linear accelerated loading test (ALT) system. In this 3D DEM model, the asphalt surface was constructed according to the random generation algorithm of irregular particles, and two important factors of temperature gradient and fatigue damage were taken into account to simulate permanent deformation, shear stresses, and strains in asphalt surface layers. Xie et al. [34] constructed a 2D DEM model of asphalt pavement to evaluate the influence of transverse crack in the bottom asphalt layer on the entire structural mechanical response, while relevant visco-elastic properties of the asphalt mixture were obtained from both laboratory tests and DEM creep tests.

Based on the basic numerical simulation and field measurement of asphalt pavement’s dynamic response characteristics, the feasibility and effectiveness of relevant newly developed sensors and devices can be evaluated and validated [35]. To validate the feasibility and accuracy of an Internet of Things (IOT) system, Huang et al. [36] compared the signals of vertical acceleration obtained from the FEM simulated results and the monitored signals of sensors during the one-third scale model mobile load simulator (MMLS3) tests, while the asphalt mixture was set as visco-elastic material in the numerical model, and its values were obtained from relevant laboratory tests. Zhao et al. [37] embedded a piezoelectric transducer model into the asphalt layers in the 2D FEM model to evaluate their synergistic performance, and the numerical results were validated by conducting rutting tests using the MMLS3 system. Shi et al. [38] proposed a wireless micro-electromechanical sensor named SmartRock to replace the traditional mechanical sensors for measuring contact stress and acceleration signals of asphalt pavement under moving loads applied by an APT device, and then the 3D FEM model was constructed and validated to together evaluate characteristics of vehicle speed. Mabrouk et al. [39] developed an FE model to simulate pavement structural response under the moving rolling weight deflectometer (RWD) loads at different vehicle speeds, and then the numerical model was validated based on the data monitored at the National Center for Asphalt Technology (NCAT) full-scale test track by a newly introduced commercial RWD device. Yan et al. [40] numerically analyzed crack damage on the acceleration response of asphalt pavement and validated the FEM model by measuring vertical acceleration signals of an in-service asphalt pavement based on a series of embedded self-developed vertical acceleration nodes. In this case, it makes sense to refine the concerned key portion on the basic analysis of dynamic response characteristics of asphalt pavement using numerical simulation combined with relevant validation by full-scale field tests.

APT methods can provide a real asphalt pavement structure, real vehicle load, and even a simulative service environment, but the high costs of field tests also limit their popular applications. The valuable measured field testing data based on APT methods are often carefully and thoroughly used to validate the feasibility and effectiveness of the corresponding numerical simulation concerning pavement design and evaluation. Attention should be paid to selecting major factors according to relevant research purposes when numerical simulation related to the APT-based field test is conducted because it is also difficult to take all factors of real asphalt pavement structure and its service conditions into account.

#### 2.1.2. Pavement Structure and Material Evaluation

It is important to conduct field tests to evaluate the effectiveness of certain newly developed materials or pavement structures [41,42,43,44] for their further application and promotion. In this case, numerical simulation can be firstly conducted to lay solid foundations for the preparation of relevant field tests and then to further explore the deep mechanical behaviors of asphalt pavement. Sun et al. [45] combined field tests using an MMLS3 system, laboratory uniaxial repeated loading penetration tests, and FEM analysis together to illustrate the mechanisms of initiation and propagation of top-down cracking in asphalt pavement. In particular, the cracking initiation and propagation process was simulated by characterizing the progress of the shear damage element area under repeated loading, in which a series of reduced parameter values of the asphalt mixture’s modulus were assigned continuously. Lu et al. [46] evaluated the influence of different saturation conditions hydromechanical performance of the polyurethane-bound permeable pavement using both FEM modeling and full-scale APT tests. During the field tests, the Falling Weight Deflectometer (FWD) method was applied to measure structural deflections, which were further used to back-calculate elastic parameters for the tire–pavement interaction model, while both pore water pressure and water distribution were measured by the embedded sensors in different structural layers [47]. Peng et al. [33] numerically evaluated the dynamic response of asphalt pavement by the DEM method, in which a damage factor D was applied in parameters of elastic modulus and viscosity for the visco-elastic material of the asphalt mixture to present the deterioration of material properties, while this kind of deterioration processes were conducted using a self-developed subroutine in PFC3D. Due to the inevitable fact of the performance deterioration of the asphalt mixture in field asphalt pavement during continuous loading by vehicle wheels, it is reasonable and necessary to reflect this deteriorating process on the numerical property parameters for asphalt materials. The deterioration of the asphalt mixture’s performance can be implemented by developing subroutines in numerical software based on certain damage mechanisms, such as aging, moisture damage, and rutting, which should be supported by serials of comprehensive laboratory mechanical tests. Caro et al. [48] combined FEM and DEM methods together to build the 2D pavement model for the evaluation of the raveling characteristics of permeable friction courses. In the study, the raveling phenomenon was assumed to occur at the asphalt mortar located at the stone-on-stone contacts, while the degenerative properties of asphalt mortars were measured experimentally and then input into a numerical model for precise calculations.

#### 2.1.3. Correlating Asphalt Materials’ Properties to Pavement Performance

Abundant data on asphalt materials’ properties and the performance of asphalt pavement have been obtained from laboratory tests, field tests, and numerical simulation in research, and it makes sense to fully utilize these data by correlating material properties and structure performance.

Walubita et al. [49,50] made efforts to correlate the high-temperature rheological properties of asphalt binders using the multiple stress creep and recovery (MSCR) tests and the rutting resistance of the asphalt mixture using the Hamburg wheel tracking (HWT) tests to the field rutting performance of in-service test asphalt pavement. Laboratory testing results were made by correlations with the database of field rutting performance using linear, power, exponential, and logarithmic functions. Similarly, Zhang et al. [51] collected rut depth data from 50 field pavement sections and cored specimens for further laboratory testing to obtain volumetric properties and HWT rutting depths, and then a predicting model for field rutting depths was built, which took pavement service age, environmental and traffic factors, and HWT rutting depth data into account. The direct regression analysis method provides a useful and applicable idea for numerical simulation to construct a correlation between asphalt material properties and asphalt pavement performance.

It is essential to evaluate the service performance status of in-service asphalt pavement using suitable non-destructive testing methods. The FWD method applied a standard 50 kN non-destructive load on asphalt pavement, and the testing point’s moduli could be back-calculated. When combined with the thickness data measured by the Ground Penetrating Radar (GPR) method and the systematically measured master curves of the asphalt mixture cored from a field road, the visco-elastic responses of asphalt pavement could be estimated [52]. Moreover, performance conditions of in-service asphalt pavement can be also evaluated and numerically analyzed based on the signals monitored by stain and stress sensors, which should be buried into different pavement layers previously.

Additionally, it is important to connect properties between different scales together, such as placing the microstructure model of the asphalt mixture into the full-scale model asphalt pavement, which can help to obtain more real characteristics for a better understanding of asphalt pavement and material performance [53]. Sun et al. [54] adopted the two-way coupled approach to take both pavement level and mixture level into account together to construct a 3D multi-scale FEM model for asphalt pavement. Pavement responses under dynamic tire load were characterized at two scales, while thermal factors, such as air temperature, solar radiation, and wind speeds, were also considered.

It is a challenge to deeply utilize measured data of asphalt pavement performance from multiple scales tests, especially for the full-scale APT tests, due to high cost. Wang et al. [55] inversely evaluated the asphalt mixture’s fundamental visco-plastic properties under the wheel load in the asphalt pavement analyzer (APA) test via numerical simulation of 3D FEM modeling, while the constructed numerical simulative test could be applied to simulating rutting tests at different scales. Huang et al. [56] compared the evaluation of pavement response and performance under different scales of APT facilities via both laboratory tests and numerical analysis with corresponding 3D FEM models, which included the full-scale ALF, MMLS3, and APA. Characteristics of several indicators such as rut depth, strain response, seismic stiffness, and contact stress were measured and numerically calculated. It was found that MMLS3 was effective in evaluating the rutting and fatigue performance of pavement materials, while APA was more efficient in ranking the rutting resistance of the asphalt mixture but was not suitable for evaluating fatigue resistance for the asphalt mixture. Khan and Tarefder [57] applied the numerical interconversion method with a 3D FEM model of asphalt pavement to convert the field sensor’s data to basic visco-elastic properties of the asphalt mixture, such as the relaxation modulus and dynamic modulus, which could be applied to assess the service condition of the asphalt pavement for preventive maintenance.

### 2.2. Interaction between Vehicle Wheel and Road Surface

#### 2.2.1. Wheel–Pavement Interaction

**Figure 4:**
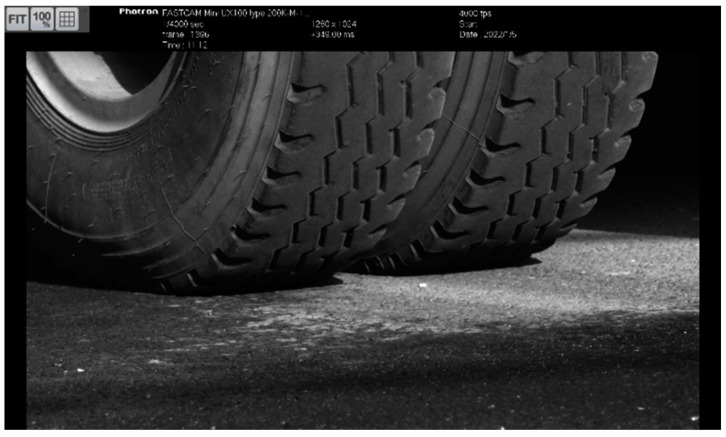
The interaction between the vehicle wheel and asphalt pavement at a vehicle speed of 20 km/h (captured by a high-speed camera, 4000 fps, 1280 × 1024 pixels, Date: 5 January 2022).

The interaction between vehicle wheels and the asphalt pavement surface, as shown in Figure 4, is essential to help to better understand the structural response features and relevant damage mechanisms. Precise FEM wheel–pavement models were often constructed to investigate contact stress distribution, road surface friction, pavement response, and so on. Ding et al. [58] built a coupled 3D tire–pavement interaction model to predict tire rolling resistance related to energy dissipation under different tire temperatures and loading conditions. Bai et al. [59] modeled the interaction between vehicle wheel and pavement using a 3D FEM model, in which both non-uniform distributed wheel–pavement contact pressure, full interfacial layer bonding conditions, and asphalt materials’ visco-elastic performance were taken into account.

It is important to take the non-uniform wheel contact pressure into account for asphalt pavement, which not only directly determines dynamic responses inside near-surface layers but also affects the design of the asphalt pavement structure [60]. Zheng et al. [61] constructed a precise 3D FEM model to analyze contact characteristics between a textured asphalt surface and a patterned wheel under dry conditions. In this study, contact pressure distribution under static loading and antilock braking system (ABS) states were discussed, respectively, while relevant indicators such as inflation pressure, wheel load, stress distribution along different paths, and contact footprint were analyzed and compared. It was found that the shoulder position and tread center area of the vehicle wheel were more susceptible to wear, and the contact pressure reached the largest values in the wheel’s center area. Wang et al. [62] numerically evaluated the dynamic response of asphalt pavement based on the input of the non-uniform tire–pavement contact stresses obtained from the indoor measurement, in which vertical pressure distribution under different rolling conditions of vehicle wheels, such as braking, free rolling, and traction, were taken into account. Liu and Al-Qadi [63] presented a deep learning method to quickly and accurately predict non-uniform tire–pavement contact stresses. Behroozinia et al. [64] discussed the wheel–pavement interaction features from the concept of intelligent wheels using a precise 3D FEM model of vehicle wheels. In this study, the influential factors of vehicle velocity, normal load, and friction coefficient on the contact patch area were analyzed, while the numerical results were validated by the acceleration signals measured by a tri-axial accelerometer attached to the inner liner of the intelligent tire. Said et al. [65] evaluated the features of the interaction between a new-generation wide-base tire and typical asphalt pavement using the FEM method and lifecycle assessment and assessed the effects of loading and environmental factors. Compared with the dual tire assembly, the wide base tire showed lager values of vertical contact force along the tire width and resulted in greater critical pavement responses.

**Figure 5:**
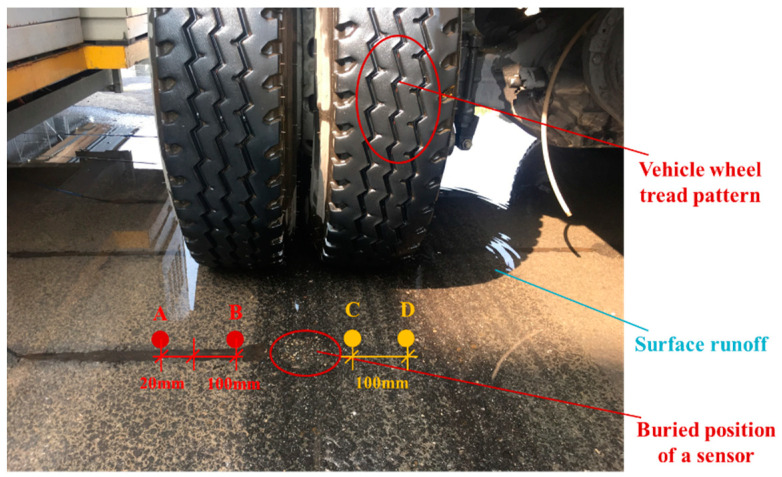
Relative position between the buried sensor and vehicle wheel.

Wheel tread patterns also affected the precision of measured signals of dynamic responses due to the confined cavity formed by wheel tread patterns and textures of the pavement surface. The authors measured dynamic pore water pressure generated in saturated asphalt pavement using the full-scale NE-ALT system [32]. As shown in Figure 5, relative positions between the buried sensor and vehicle wheel could be adjusted by controlling the wander parameter of the NE-ALT system’s vehicle simulator. Test point A was 20 mm away from the outside edge of the left wheel and point B was located at the middle position of the contact area between the left wheel and pavement, while both test points C and D were located in the wheel–pavement contact area, and their distance was 100 mm. It was found that the difference value of measured pore water pressure magnitudes between points A and B reached 93.7%, while this difference value between points C and D was up to 86.1%. The conclusion of the full-scale APT tests is one of the pieces of evidence that shows the great influence of wheel tread patterns on the mechanical response of asphalt pavement, which may also affect the validation of numerical simulations. It also indicates the importance of controlling the path of the trial vehicle wheels and ensuring enough parallel trials.

#### 2.2.2. Hydroplaning Phenomenon

**Figure 6:**
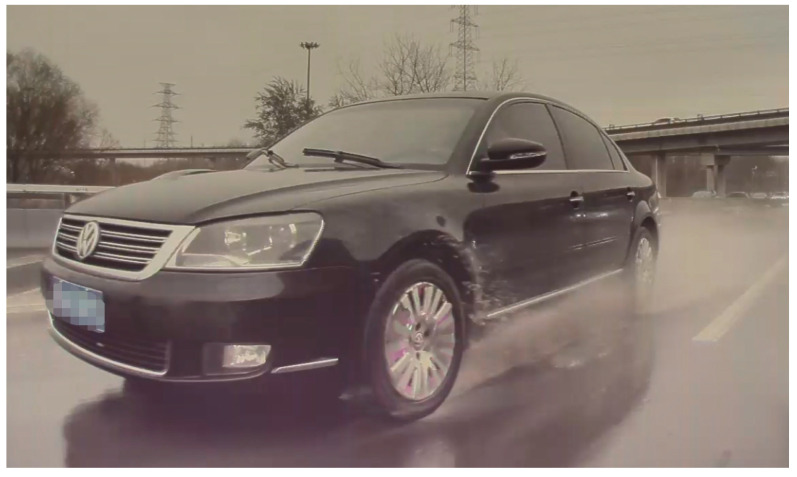
The wheel–pavement–water interaction.

The complicated service environmental factors that asphalt pavement has to face, such as rainfall, freeze–thaw, and ultraviolet radiation, will challenge its performance and service life. When a vehicle drives in rainy weather, as shown in Figure 6, the interaction between wheels, asphalt pavement, and the water film will cause driving risks and adverse mechanical effects on pavement structure [31,32], while a typical hydroplaning phenomenon may happen if the driving speed is quite fast with a certain water film thickness. Peng et al. [66] built a 3D FEM wheel–pavement–water model to numerically analyze the maximum safe driving speed on wet horizontal pavement curves, while pavement material properties and drainage data were input to the model. The available wheel–pavement frictional resistance was first calculated and then combined with centrifugal force to analyze the wheel’s skidding potential. Serials of maximum safe vehicle speeds were thus numerically calculated corresponding to relevant parameters, such as water film thickness, curve radius, and the curve’s superelevation rate. Ding and Wang [67] compared hydroplaning speeds of different types of vehicle wheels using 3D FEM fluid–structure interaction models, in which several indicators such as water flow into wheel contact path, contact force, water film thickness vs. drainage path length, and hydroplaning speed were comprehensively analyzed. It was found that hydroplaning potential increases with the increase in water film thickness, while high wheel load or high wheel inflation pressure positively increases hydroplaning speed. Zhu et al. [68] analyzed the skid resistance performance of an aircraft on a wet rough runway using a 3D prototype finite element co-simulation. In this study, a precise 3D patterned vehicle wheel and textured pavement surface FEM model were established for hydroplaning characteristics evaluation, and the numerical results were validated by the field measurement of slip ratio, velocity, and friction coefficient by a force transducer mounted on a trailer’s wheel. It was found that the increase in water film thickness and grounding speed caused the attenuation of the friction coefficient of a runway, which easily caused aircraft overrun. Gerthoffert et al. [69] built a small-scale cofferdam on the pavement surface with continuous watering by a hose, and then directly measured pavement friction with surface runoff using the brush method.

The skid resistance performance of the surface layer Is demanded to be measured and checked according to the specifical standard when pavement and the surface material are designed. However, the reduced frictional properties of surface material related to accumulated water film drew limited attention during pavement and material design periods. It is difficult to directly simulate the experimental conditions related to the hydroplaning phenomenon, which mainly include thick water depth and high driving speed, but systematic numerical simulation together with a few laboratory tests can help to explore the characteristics of this phenomenon. For example, the skid resistance of the surface asphalt mixture with different water film thicknesses can be directly measured, which may be an essential supplement for the asphalt mixture design. The water flow field characteristics and friction features of the road’s surface layer can be also obtained indoors using a specific wheel tracking device with low driving speeds, which can be applied to verify the effectiveness of the numerical simulation model, and the verified model can further predict performance related to high driving speeds.

Water film thickness on the pavement surface is one of the important parameters for the skid resistance evaluation of driving vehicles, and the features of which are the essential foundation for further numerical simulation of hydroplaning and need to be appropriately characterized. Schulz et al. [70] derived the theoretical hydrodynamic model for water films based on the fluid–surface interaction considering relevant factors, such as flow field distribution, rain intensity, and pavement texture, while the model was validated by field experimental data. Luo et al. [71] constructed full-scale road surface models to directly observe water film features under different parameter combinations, including rainfall intensity, slope, and texture of the asphalt mixture types, while several regression models were built to predict water film thickness. Luo et al. [72] developed an analytical water film depth model that included features of pavement texture, slope, permeability, and rainfall coefficient, while a new device named the Rainwater Level Measuring Instrument (RLMI) was adopted to acquire field rainfall data and water film depth data for model validation. Further, Luo and Li [73] adopted a 3D line scanning laser system based on the survey vehicle to obtain features of pavement rutting and texture with high precise and efficiency, which helped to improve the prediction of the proposed model for water file thickness. Geng et al. [74] built a numerical model of straight line segment asphalt pavement runoff based on the hydrodynamic method of 2D shallow water equations, while the model was verified by field tests using the short-range telemetry technology based on the narrow-wave infrared spectroscopy. The monitoring system in this study was mounted in mid-air on a road shoulder with the support of a bracket, which looked like a street lamp.

## 3. Macro- and Mesoscale Properties Modeling on the Asphalt Mixture and Its Design

### 3.1. Mechanical Behaviors Evaluation on the Asphalt Mixture

Compared with traditional mechanical tests for the asphalt mixture, such as the semi-circular bending (SCB) test, the indirect tensile test (IDT), and the three-point bending test, relevant virtual tests conducted by numerical simulation can provide more characteristics and details inside specimens, which may be not easy to be obtained from laboratory tests. After being validated by laboratory mechanical tests, numerical models can be fully used to further explore and predict relevant mechanical performance in several additional virtual testing conditions, which mainly include environmental factors and loading parameters. Relevant algorithms are essential to be proposed to build a virtual specimen of the asphalt mixture to simulate characteristics of the real specimen, which mainly include aggregate particle features, gradation, distribution, and air void. In particular, digital image correlation (DIC) technology [75] is a useful method to measure the strain field characteristics on the specimen with a typical flat surface for the asphalt mixture, which can provide more mechanical features and can be directly compared with the strain distribution on digital specimen’s surface in the numerical models.

For the SCB test, Zhao et al. [76] raised an image selection process method, which included two important parameters, the aggregate content indicator (ACI) and the aggregate distribution indicator (ADI), to enhance the accuracy of the 2D FEM simulation on SCB performance for the asphalt mixture. This image selection process method was also applied by Zhao et al. [77] to improve the accuracy of 2D FEM modeling for the asphalt mixture in IDT tests. Song et al. [78] built a 2D extended finite element model (XFEM) of the SCB test for the asphalt mixture to capture its fracture behaviors. In this study, stress distribution at different stages during SCB loading was obtained, which could help to better understand the fracture characteristics of the entire specimen and the local stress features around different aggregate particles.

For the IDT test, Chang et al. [79] built a DEM model to characterize the distribution features of force chains inside the asphalt mixture. Liu et al. [80] built 2D FEM models of the asphalt mixture microstructures based on CT reconstruction to evaluate the influence of different fillers on the mechanical responses of mixture specimens in IDT tests, which included load-bearing capacity, von Mises stress, and creep strain.

For the three-point bending test, Bai and Wang [81] established a random aggregate model based on the DEM method to reflect the heterogeneity of the asphalt mixture, which could show the aggregate’s features of sphericity, angularity, roughness, and gradation. In this study, the three-point bending test was numerically analyzed to explore the expansion mechanism of the I-type crack of the asphalt mixture. Nian et al. [82] adopted the PFC2D method to construct mesoscopic models of the asphalt mixture in IDT and three-point trabecular bending tests to evaluate its low-temperature anti-cracking performance. In this study, the generation of 2D DEM specimens was based on the aggregate distribution features of real asphalt mixture specimen slices. Teng et al. [83] established a random heterogeneous FEM model of the asphalt mixture, which considered features of aggregate gradation and air void to numerically conduct three-point bending tests for low-temperature cracking characteristic evaluation.

Moreover, several other types of mechanical tests [84,85,86,87,88] and comprehensive performance inspections [89,90,91,92,93,94] can be also conducted by numerical methods. Papagiannakis et al. [95] built a DEM model of the asphalt mixture using its microstructure obtained by CT technology to evaluate the plastic deformation behavior of the asphalt mixture subjected to the loading of the asphalt mixture performance test (AMPT). The numerical values of flow number and their corresponding plastic strain were further compared with laboratory rutting tests with the Hamburg wheel tracking test device. Peng et al. [96] investigated the influence of void features on uniaxial penetration strength (UPS) for the asphalt mixture based on the CT reconstruction method and 3D DEM modeling. Ge et al. [97] built the 3D DEM model of a trapezoidal specimen for the asphalt mixture in the two-point bending test based on the 3D aggregate morphological characteristics obtained using laser scanning, and then numerically evaluated the complex modulus utilizing the contact dynamic s method. Yuan et al. [98] built a 3D DEM model in uniaxial compression loading for a large stone porous asphalt mixture based on the rebuilt irregular aggregate particles using CT technology, while contact characteristics such as coordination number, contact point, and energy were obtained based on the flat-joint contact model, and the compressive strength was also discussed. Ji et al. [99] evaluated the rutting behavior of the asphalt mixture modified by Direct Coal Liquefaction Residue (DCLR) using a 2D FEM modeling. Visco-elastic parameters in this study were obtained from dynamic creep test and time-hardening power creep law, while the influence of temperature and tire pressure were taken into account. Sadeghnejad et al. [100] constructed the 2D FEM model to evaluate the rutting behavior of a glasphalt mixture considering the impact of temperature and stress, while the creep power low model was applied to evaluate the visco-elastic and visco-elasto-plastic performance of the asphalt mixture, and the numerical results were finally validated by conducting wheel track tests.

### 3.2. Proportion Design of the Asphalt Mixture

With the development of advanced techniques, such as CT and the Aggregate Image Measurement System (AIMS) [101], it is possible to reconstruct 3D digital aggregate particles with morphological properties [102,103,104], which makes it possible to directly conduct a virtual design of the asphalt mixture. During the virtual design process, several basic parameters, such as aggregate gradation, spatial distribution, orientation, shape, angularity, texture, aggregate content, asphalt content, and air void content, will undoubtably affect the macroscale performance of the entire asphalt mixture and the microscale mechanical response inside a sample.

**Figure 7:**
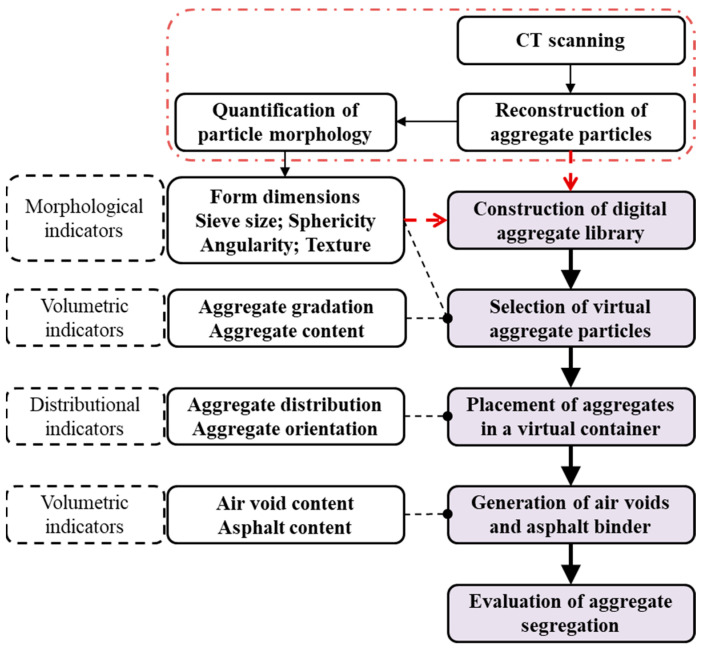
Typical procedures of virtual specimen design for the asphalt mixture [105].

Jin et al. [105] designed and constructed a 3D virtual asphalt mixture based on a digital aggregate library composed of 3000 reconstructed particles of real aggregates, and their morphological properties were also quantified. Comparing volumetric and morphological assignments with the aggregate component of the asphalt mixture, a serial of virtual aggregates was selected from the digital library, which were then determined with their positions and orientations based on their distributional assignment inside a specimen. The microstructure of a virtual mixture was thus obtained after the determination of the components of asphalt mastics and air voids, while the virtual specimen was then evaluated by a FEM IDT test. The procedures of virtual specimen design for the asphalt mixture are summarized in Figure 7. Considering the high cost of reconstruction of the 3D mixture based on the characteristics of realistic aggregate component properties, Manrique-Sanchez et al. [106] generated a 2D DEM specimen of the asphalt mixture using gravimetric methods, while the DEM sample was then implemented into ABAQUS to evaluate its mechanical response in the dynamic axial modulus test. Li and Wang [107] adopted the DEM method to simulate the design process of constructing air voids and aggregate skeleton structures for the asphalt mixture with different gradations. In this study, several important factors, such as interaggregate contacts and local stability for aggregate particles and visco-elastic contact between asphalt binders and aggregates, were taken into account. Exploring features of indicators, such as density, air voids, and flow of the asphalt mixture, the DEM compaction processes in the Superpave Gyratory Compaction (SGC) model were simulated, and the effect of compaction temperature was further discussed.

Ren et al. [108] assessed the influence of different aggregate particle sizes on the air void ratio and macroscopic aggregate bearing capacity of a porous asphalt mixture (PAM). Considering mesoscopic aggregate contact force, the gradation of the PAM was optimized based on a DEM method. Liu et al. [109] characterized the field compaction process of an epoxy asphalt mixture using the DEM method, while the motion and contact force of particles under compaction loads were discussed and the effect of mixture agglomeration on compaction degree was analyzed. Chang et al. [110] reconstructed coarse aggregate based on laser scanner technology and then simulated the compaction process in the SGC model using the DEM method. In this study, the influence of aggregate segregation on mechanical properties of the asphalt mixture, such as compressive strength in a uniaxial compression test and cohesive force and internal friction angle in a uniaxial penetration test, were evaluated.

### 3.3. Components’ Properties of the Asphalt Mixture

The components of the asphalt mixture, such as the asphalt binder (also including mortar and mastic), aggregate, and fiber, play important roles in the various performances of the entire mixture, the properties of which can be analyzed by a numerical simulation.

For the asphalt binder, Ziade et al. [111] evaluated the rheological behavior of asphalt materials, which included the asphalt binder, mastic (mixed with mineral fillers passing through a 0.063 mm sieve), and mortar (mixed with mineral filler and fine aggregate smaller than a 0.25 mm sieve) using the laboratory frequency sweep test, analytical approach, and 3D FEM modeling. In this study, a virtual DSR test could be conducted to evaluate stress and strain distribution in the digital sample’s microstructure. Giancontieri et al. [112] constructed a computational fluid dynamics (CFD) model in ANSYS software of version 17 to simulate the rotational viscosity test for modified asphalt binders, e.g., a recycled tire rubber modified binder, while indicators of shear rate, viscosity, and particle distribution were numerically analyzed by a standard Brookfield smooth spindle, and an improved dual helical ribbon was compared. Ye et al. [113] numerically evaluated the process during the asphalt penetration test using the FEM method. Comparing the penetration values vs. time between numerical results and laboratory measurements with the help of a high-speed camera, the time–history curves for displacement, strain, and stress at the monitoring points were numerically analyzed.

For other components inside the asphalt mixture, Wu et al. [114] conducted 3D FEM modeling on the Micro-Deval test to evaluate the wear properties of aggregates. Tan et al. [115] focused on the aggregate-to-aggregate contact issue and quantitively evaluated the effect of the contact zone on the visco-elastic performance of the asphalt mixture using the 3D FEM microstructural model. Cheng et al. [116] investigated the distribution of basalt fibers inside asphalt mortar using 3D FEM modeling and then validated the numerical results by CT scanning and reconstruction. In this study, the virtual three-point bending test was conducted to evaluate the relationship between uneven distribution features of basalt fibers and the flexural tensile performance of the asphalt mixture.

## 4. Microscale Features Modeling on the Asphalt Mixture

### 4.1. Performance Modeling Based on a Two-Dimensional Cross-Section of the Asphalt Mixture

The digital image processing (DIP) method is usually adopted to analyze the 2D scanned image of an asphalt mixture sample cross-section for the acquisition of internal structural characteristics of the asphalt mixture [117,118,119]. The 2D images can be directly scanned using a digital camera or selected from a serial of images obtained by CT technology. On the basis of the 2D microstructural features of the asphalt mixture, it is possible to numerically analyze the interlocking mechanical behaviors between aggregate particles inside the mixture using the FEM and DEM methods. Zhao et al. [120] constructed a 2D DEM model to simulate the three-point bending test for the asphalt mixture based on the image recognition of a real asphalt mixture’s flat surface. During the loading process, meso-crack formation and propagation between aggregate particles were discussed, and so were the distribution and transmission of stress and the evolution of the displacement field. It was found that the DEM method obtained internal stress and displacement of the asphalt mixture, which could help to reveal the evolution of mechanical behavior between particle flows. Zhang et al. [121] generated the 2D FEM virtual specimen of the asphalt mixture based on the 2D slices of laboratory samples’ digital images, while modulus results of the digital mixture and trial samples were compared and analyzed. Liu et al. [122] built the 2D FEM model for the asphalt mixture sample compacted by a newly developed laboratory Aachen compaction using the DIP method, while virtual IDT tests were conducted on field cores, Aachen specimens, and Marshall specimens for the purpose of verifying the effectiveness of the Aachen compaction method.

### 4.2. Performance Modeling of the Asphalt Mixture Based on X-ray Computed Tomography Technology

**Figure 8:**
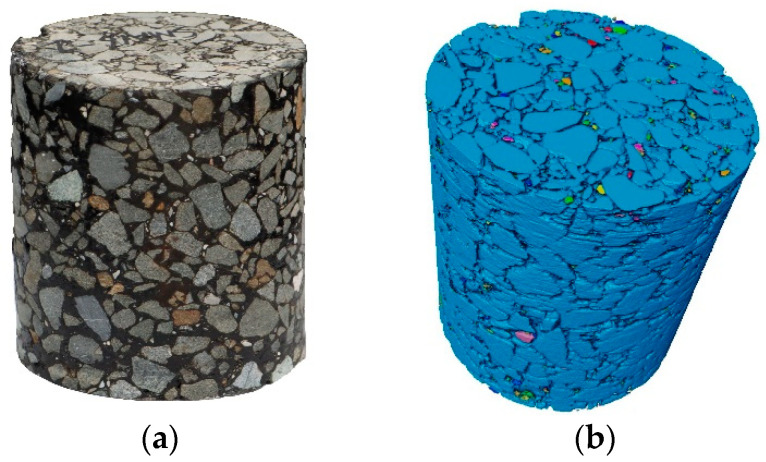
CT scanning and 3D reconstruction on an HMA specimen: (**a**) the real specimen, (**b**) the 3D virtual specimen.

X-ray CT technology is usually adopted to scan a specimen of an asphalt mixture slice by slice along its height direction [123,124,125,126], and the obtained images can be analyzed using the DIP method to reconstruct the 3D microstructure of the asphalt mixture for further investigation [127,128], as shown in Figure 8. The spatial distributions of aggregates, air voids, and even water flow can be separately divided from an entire mixture sample, which makes it possible to illustrate mechanisms of multiple damages for asphalt materials, such as moisture damage, rutting, cracking, and clogging.

For water flow characteristic evaluation, Ma et al. [74] evaluated internal water flow characteristics inside permeable pavement considering the clogging factor, which decreases the content of air voids. In this study, a 3D FEM model of a permeable asphalt mixture was generated based on the structural reconstruction of the CT scanning images, while spatial distributions of aggregate skeletons, air voids, and seepage flows were then obtained and analyzed. It was found that internal flow nearly disappeared if the porosity decreased by less than 15%, while clogging also affected surface hydrodynamic pressure distribution, which was directly related to the hydroplaning phenomenon. Chen et al. [129] used CT scanning images to reconstruct the 3D pore structure of the open-graded friction course (OGFC) specimen using the DIP method, while pore features such as length, curvature, and hydraulic diameter were calculated and analyzed. It was found that the water flow rate in the horizontal direction was larger than that in the vertical direction if gravity was not considered. Meng et al. [130] investigated the preferential path of water flow inside a permeable asphalt mixture using the topological network model, while the fluid flow characteristics were evaluated using a 2D FE model. Ghauch et al. [131] constructed a 3D FEM model of the asphalt mixture based on CT scanning technology and then numerically investigated the effect of moisture on the microscale and macroscale responses of the asphalt mixture, while the cohesive and adhesive damage features could be thus assessed.

Lv et al. [132] evaluated the three-stage damage evolution of the asphalt mixture in the wet HWT test using CT technology, which mainly included post compaction, the creep stage, and the strip stage, while CT images in the top view and side view of the samples were compared and analyzed. The evolution of the 3D air void distribution with loading passes in the HWT tests was investigated by direct intuitive image analysis. Lövqvist et al. [133] generated a 3D FEM microstructural model for the asphalt mixture based on the analyzed CT scanning images to discuss freeze–thaw damage mechanisms, while parameters of moisture infiltration and temperature and the mechanical properties of asphalt binders and interfacial features were taken into account. You et al. [134] built a 3D FEM CT-based model of the asphalt mixture to predict its thermo-mechanical responses, while comprehensive material properties such as visco-elastic, -plastic, and -damage constitutive models were considered.

## 5. Molecular-Scale Behavior Modeling on Asphalt Materials

### 5.1. MD Simulation on the Asphalt Binder and Mineral Aggregate

A molecular dynamics (MD) simulation is a good numerical method that can help to explore fundamental characteristics of asphalt materials such as asphalt binders, mineral aggregates, and their relevant interface interaction from a molecular scale, while thermodynamic and mechanical behaviors can be also analyzed.

For the asphalt binder, the influence of its different types of components on its entire presented properties can be well evaluated using MD simulation, while the interaction features between water molecules and asphalt components can also be assessed [5,8,135]. Du et al. [136] analyzed the diffusion and structural properties of moisture (water molecules) in both the neat asphalt binder and asphalt mastic using MD modeling, while microstructural changes of indicators, such as free volume and hydrogen bond formation, during the diffusion process were discussed. It was found that moisture diffusion in asphalt materials was controlled by the free volume and the cohesion property among asphalt chains. As paraffin showed a negative effect on the performance of asphalt binders, Qu et al. [137] evaluated the influence of its different contents on the microscopic properties of asphalt binders using MD simulation. The evaluated indicators mainly included density, Young’s moduli, Poisson’s ratio, bulk moduli, and shear moduli, while the self-healing behavior of asphalt binders was also discussed. It was found that the existence of paraffin significantly reduced both low-temperature and high-temperature stabilities, mechanical properties, and the self-healing rate for asphalt binders, which indicated that the existing content of paraffin inside asphalt binders should be controlled at a minimum amount. In order to systematically evaluate the self-healing behavior of an asphalt binder, Qu et al. [138] separated it into six fractions by MD modeling, which included saturates, monoaromatics, diaromatics, polyaromatics, resins, and asphaltenes, while several influential factors, such as crack width, temperature, molecular aggregation state, and aggregate, were taken into account. It was found that graphene exhibited some positive impacts on the self-healing process of asphalt binders.

MD simulation can effectively evaluate the improving effects of several additives on the performance of asphalt binders, which provides an idea to help explore damage mechanisms related to various environment-induced aging. Peng et al. [139] assessed the effect of waste polyethylene (WPE) on asphalt binder’s performance before and after oxidative aging using the MD simulation, while several indicators such as density, viscosity, glass transition temperature, and cohesive energy density (CED) were taken into account. During oxidative aging of the asphalt binder, it was found that WPE could reduce viscosity change and CED value, while it could also improve the self-healing ability to a certain degree. Cao et al. [140] discussed the effect of photocatalyst titanium dioxide (TiO_2_) on asphalt binder’s chemical structure and properties using both MD simulation and laboratory tests, such as AFM and FTIR.

For mineral aggregate, Zhu et al. [141] evaluated the reinforcement of mineral fillers on the structural, thermodynamic, and mechanical properties of asphalt mastic using the MD simulation. An asphalt–silica nanoparticle composite system was established to simulate asphalt mastic, and its structural properties of free volume and radial distribution function were then calculated. It was found that silica particles decreased the interaction between asphalt molecules and increased free volumes in the configuration [142].

### 5.2. MD Simulation on Features of the Asphalt–Aggregate Interface

Interfacial behaviors between asphalt and aggregate play an important role in the mechanical integrity of the asphalt mixture [143,144,145]. Not only trial methods concerning surface free energy theory but also numerical MD simulation can also quantify cohesive bonding between asphalt binders and aggregates, while the later can be adopted to deeply explore thermodynamic features of the asphalt–aggregate interface. In particular, no matter whether in laboratory inspecting research or numerically virtual testing studies, the mode of the direct pull-off test is often applied to evaluating asphalt–aggregate interface characteristics.

Zhai et al. [146] discussed the effect of aging and moisture on thermodynamic properties and failure behaviors of the asphalt–aggregate interface using MD simulation, while thermodynamic parameters of surface free energy, cohesive work, and adhesion work were numerically analyzed. In particular, a virtual pull-off test was conducted to analyze interfacial failure patterns and mechanisms. It was found that oxidative aging reduced thermodynamic properties but enhanced the potential and nonbonded energy of asphalt binders. The numerical results concerning moisture damage for different combinations between asphalt binders and mineral aggregates with different types of lithology were consistent with relevant trial results obtained from Sessile Drop tests [147]. Du et al. [148] conducted the virtual pull-off test to numerically analyze interfacial strength between asphalt and aggregate based on MD simulation, while the mechanical and deformation behavior of the asphalt–aggregate interface under tensile stress could be well evaluated. Moreover, Du et al. [149] evaluated the effect of aggregate surface roughness on the interfacial properties of the asphalt–aggregate MD model. Zhang et al. [150] numerically evaluated the coupling effects of moisture and temperature on interfacial adhesion features between rubberized asphalt binders and steel slag, while the molecular scale interface interaction was compared with a macroscopic direct tensile bonding failure test and followed by correlation analysis. It was found that a rising temperature drove water molecules to gather close to the steel slag surface but drove asphalt molecules away from steel slag molecules, which indicated a high risk of moisture damage. Wang et al. [151] further discussed the effect of mineral composition on the interaction of the rubberized asphalt–aggregate interface.

## 6. Nanoscale Characteristics Modeling on the Asphalt Binder

The bee structure of the asphalt binder can be characterized based on the atomic force microscopy (AFM) method at the nanoscale [152,153,154], which mainly includes bee, peri, and interstitial phases. The captured AFM image of the asphalt binder helps to build the numerical model for the asphalt binder that can be adopted to evaluate comprehensive nanoscopic performance [155], which makes it possible to investigate aging damage mechanics induced by service environments.

Shan et al. [156] constructed the 2D FEM model of the asphalt binder at the nanoscale based on the measured morphology results from AFM tests. In this study, a self-developed in situ tensile loading device for the AFM method was adopted to measure the stress distribution of the asphalt binder sample’s surface under tensile loading, and the results of laboratory tests and numerical simulation were combined together to illustrate the damage mechanism of the asphalt binder. Du et al. [157] constructed 2D FEM models of asphalt binders with different microstructural phases based on AFM images, while this model was applied to simulate micromechanical responses of asphalt binders with different bee structures. It was found that the increase in the peri phase content would increase the sample’s load-bearing capacity, which also made the distribution of tensile strain become more homogeneous.

## 7. Discussion

On the basis of validation by experiments with a few limited numbers, multi-scale numerical simulation can help to obtain abundant material properties and structural performance of asphalt pavement. Structural dynamic responses of asphalt pavement are commonly simulated and analyzed, while the full-scale APT test method is often applied to validate the feasibility and effectiveness of the generated numerical model using the valuable measured field-testing data with a suitable cost. The concerned portion can be refined and reflected on the numerical pavement model, such as relevant newly developed sensors, while numerical simulation of the structural response of asphalt pavement can help to verify the possibility and effectiveness of their applications. Compared with FEM modeling on pavement responses, DEM modeling takes features of aggregate particles into account, but it may consume many more computations. When numerically evaluating the performance deterioration of the asphalt mixture during continuous loading by vehicle wheels, the decline evolution of material parameter values should be noticed and exhibited, which is usually implemented by developing subroutines. It is a challenge to present multiple performance deterioration mechanisms such as ageing, moisture damage, and rutting into numerical simulation simultaneously. Efforts have been made to correlate asphalt material properties to pavement performance, such as direct regression analysis and reverse derivation from pavement mechanical behaviors to material characteristics, which are beneficial for material design and relevant performance prediction.

Precise FEM wheel–pavement models are often constructed to investigate characteristics of wheel–pavement interaction, but the wheel tread patterns, which result in the non-uniform wheel contact pressure, should be taken into account carefully if necessary. Considering the water factor of the wheel–pavement interaction, three aspects of seepage flow inside saturated pavement, flow features inside wheel tread patterns, and surface runoff distribution have been investigated individually in several research studies. It makes sense to unify these three aspects together to make a comprehensive analysis, which can help to understand both the hydroplaning phenomenon and the moisture damage mechanism induced by dynamic water pressure. More field studies need to be conducted in the future to validate the numerical model of the wheel–pavement–water interaction. It is essential and meaningful to pay more attention to considering the reduced frictional properties and skid resistance performance of surface materials coupled with water and vehicle loading within the pavement design period.

To simulate macroscale mechanical properties of the asphalt mixture, typical virtual tests such as the SCB test, IDT test, and three-point bending test have been conducted and analyzed using FEM and DEM modeling, which can provide more characteristics and details inside the specimen. Relevant algorithms are essential to be proposed to build a virtual specimen of the asphalt mixture to simulate characteristics of a real specimen, which mainly include aggregate particle features, gradation, distribution, and air void. DIC technology is often adopted to measure the strain field characteristics on the specimen with a typical flat surface, which can be applied to validate the effectiveness of the virtual tests mentioned above. Efforts can be made to simulate more types of complicated mechanical tests in the future.

The development of advanced techniques, e.g., CT scanning technology, meso- and microscale features of the asphalt mixture and its components, can be numerically analyzed using FEM and DEM modeling. Based on the 2D microstructure of the asphalt mixture, it is possible to numerically analyze the interlocking mechanical behaviors between aggregate particles inside the mixture specimen. In the 3D reconstructed asphalt mixture specimen, the spatial distributions of aggregates, air voids, and even water flow can be separately divided from an entire mixture sample, which makes it possible to illustrate mechanisms of multiple damages for asphalt materials, such as moisture damage, rutting, cracking, and clogging. On the basis of the reconstruction of 3D digital aggregate particles with morphological properties, it is possible to conduct a virtual proportion design for the asphalt mixture, while the compaction process can be also simulated using the DEM method. However, most attention has been paid to aggregate skeletons in a digital specimen of the asphalt mixture, but the unique features of other components, such as asphalt mortar or asphalt mastic, are often neglected. MD simulation can help to explore the fundamental characteristics of asphalt binders, mineral aggregates, and their relevant interface interaction from a molecular scale, while thermodynamic and mechanical behaviors can be also analyzed. The captured AFM image of the asphalt binder helps to build the numerical model for the asphalt binder that can be adopted to evaluate comprehensive nanoscopic performance, which makes it possible to investigate aging damage mechanics induced by service environments. In particular, the concept of the material genome, which gathers features of material property and structural performance, is essential to be introduced to numerical simulation for asphalt material design and pavement structural performance prediction.

## 8. Conclusions and Outlook

This study reviewed the research concerning aspects of the design, characterization, and prediction of performance for asphalt materials and asphalt pavement based on multi-scale numerical simulations. Based on the systematical discussions above, the following recommendations are proposed for future investigations on numerical analysis concerning performance design and prediction of asphalt materials and asphalt pavement.

(a) Investigating a certain failure mechanism of asphalt materials using multi-scale numerical simulation systematically.
(b) Taking multiple damage mechanisms such as aging, rutting, and moisture damage into account simultaneously to simulate the complex deterioration of asphalt material properties.
(c) Conducting more field tests to support the research on numerical simulation of the wheel–pavement–water interaction.
(d) Constructing databases concerning asphalt material properties and pavement structural behaviors for the purpose of numerically analyzing material design and performance prediction from the concept of material genome.

## Data Availability

Data are contained within the article.

## References

[1] Shanbara H., Ruddock F., Atherton W. (2018). Predicting the rutting behaviour of natural fibre-reinforced cold mix asphalt using the finite element method. Constr. Build. Mater..

[2] Bratu C., Ciobanu V., Derczeni R., Salca E. (2020). Study on the forest road pavements reinforced with geogrids by using the 2D FEM method. Road Mater. Pavement Des..

[3] Sun Y., Guo R., Gao L., Wang J., Wang X., Yuan X. (2019). Study on dynamic response characteristics of saturated asphalt pavement under multi-field coupling. Materials.

[4] Alvarez A., Mora J., Espinosa L. (2018). Quantification of stone-on-stone contact in permeable friction course mixtures based on image analysis. Constr. Build. Mater..

[5] Zadshir M., Oldham D., Hosseinnezhad S., Fini E. (2018). Investigating bio-rejuvenation mechanisms in asphalt binder via laboratory experiments and molecular dynamics simulation. Constr. Build. Mater..

[6] Kazmee H., Tutumluer E., Beshears S. (2017). Using accelerated pavement testing to evaluate reclaimed asphalt pavement materials for pavement unbound granular layers. J. Mater. Civ. Eng..

[7] Król J., Khan R., Collop A. (2017). The study of the effect of internal structure on permeability of porous asphalt. Road Mater. Pavement Des..

[8] Yaphary Y., He M., Lu G., Zou F., Liu P., Tsang D., Leng Z. (2023). Experiment and multiscale molecular simulations on the Cu absorption by biochar-modified asphalt: An insight into removal capability and mechanism of heavy metals from stormwater runoff. Chem. Eng. J..

[9] Leon L., Gay D. (2019). Gene expression programming for evaluation of aggregate angularity effects on permanent deformation of asphalt mixtures. Constr. Build. Mater..

[10] Maia R., Costa S., Cunto F., Branco V. (2021). Relating weather conditions, drivers′ behavior, and tire-pavement friction to the analysis of microscopic simulated vehicular conflicts. J. Transp. Eng. Part B Pavements.

[11] Fang M., Park D., Singuranayo J., Chen H., Li Y. (2019). Aggregate gradation theory, design and its impact on asphalt pavement performance: A review. Int. J. Pavement Eng..

[12] Schuck B., Teutsch T., Alber S., Ressel W., Steeb H., Ruf M. (2021). Study of air void topology of asphalt with focus on air void constrictions—A review and research approach. Road Mater. Pavement Des..

[13] Plessis A., Boshoff W. (2019). A review of X-ray computed tomography of concrete and asphalt construction materials. Constr. Build. Mater..

[14] Wang H., Ding H., Feng B., Shao L., Qu X., You Z. (2020). Advances on molecular simulation technique in asphalt mixture. J. Traffic Transp. Eng..

[15] Chen Z., Pei J., Li R., Xiao F. (2018). Performance characteristics of asphalt materials based on molecular dynamics simulation—A review. Constr. Build. Mater..

[16] Tan Y., Li G., Shan L., Lv H., Meng A. (2020). Research progress of bitumen microstructures and components. J. Traffic Transp. Eng..

[17] Han Z., Cong P., Qu J. (2022). Microscopic experimental and numerical research on rejuvenators: A review. J. Traffic Transp. Eng. (Eng. Ed.).

[18] Dhakal N., Elseifi M., Al-Qadi I., Rupnow T. (2021). Effect of pavement responses on fatigue cracking and cement-treated reflective cracking failure mechanisms. J. Transp. Eng. Part B Pavements.

[19] Assogba O., Tan Y., Zhou X., Zhang C., Anato J. (2020). Numerical investigation of the mechanical response of semi-rigid base asphalt pavement under traffic load and nonlinear temperature gradient effect. Constr. Build. Mater..

[20] Estaji M., Coleri E., Harvey J., Butt A. (2021). Predicting excess vehicle fuel use due to pavement structural response using field test results and finite element modelling. Int. J. Pavement Eng..

[21] Saliko D., Erlingsson S. (2021). Damage investigation of thin flexible pavements to Longer Heavier Vehicle loading through instrumented road sections and numerical calculations. Road Mater. Pavement Des..

[22] Sok T., Kim Y., Lee S. (2021). Numerical evaluation of built-in temperature distribution effects on stress development in concrete pavements. Road Mater. Pavement Des..

[23] Okte E., Al-Qadi I. (2022). Prediction of flexible pavement 3-D finite element responses using Bayesian neural networks. Int. J. Pavement Eng..

[24] Onur O., Hasan Y., Nilufer O., Turan O. (2022). Evaluation of mechanical properties and structural behaviour of concrete pavements produced with virgin and recycled aggregates: An experimental and numerical study. Int. J. Pavement Eng..

[25] Kabir R., Hiller J. (2021). Numerical analyses of rigid and flexible pavements responses under heavy vehicles’ loading. Road Mater. Pavement Des..

[26] Gabriel B., Mansour E., Sebaaly P., Ji R., Garg N. (2021). Instrumented flexible pavement responses under aircraft loading. Int. J. Pavement Eng..

[27] Yang Z., Wang Z., Chen E., Si C., Wang X. (2019). Dynamic response analysis of vehicle-load on asphalt pavement based on discrete element method. China J. Highw. Transp..

[28] Neves J., Freire A., Qamhia I., Al-Qadi I., Tutumluer E., Chastre C., Neves J., Ribeiro D., Neves M.G., Faria P. (2023). Full-scale accelerated pavement testing and instrumentation. Advances on Testing and Experimentation in Civil Engineering.

[29] Plessis L., Ullo A., Harvey J., Coetzee N. (2018). Accelerated pavement testing efforts using the heavy vehicle simulator. Int. J. Pavement Res. Technol..

[30] National Center for Materials Service Safety, University of Science and Technology Beijing (2023). Special-Regional Environment Test Facility. http://en.ncms.ustb.edu.cn/Plantconstruction/samtffse/.

[31] Wang W., Yan G., Zhao K., Wang L. (2022). Numerical simulation and experimental measurements of dynamic responses of asphalt pavement in dry and saturated conditions under full-scale accelerated loading. Appl. Sci..

[32] Wang W., Zhao K., Li J., Luo R., Wang L. (2021). Characterization of dynamic response of asphalt pavement in dry and saturated conditions using the full-scale accelerated loading test. Constr. Build. Mater..

[33] Peng Y., Xia S., Xu Y., Lu X., Li Y. (2022). Mechanical response of asphalt surfaces under moving traffic loads using 3D discrete element method. J. Transp. Eng. Part B Pavements.

[34] Xie S., Yi J., Wang H., Yang S., Xu M., Feng D. (2022). Mechanical response analysis of transverse crack treatment of asphalt pavement based on DEM. Int. J. Pavement Eng..

[35] Montoya A., Jagtap P., Papagiannakis A., Dessouky S., Walubita L. (2020). Numerical study on design and installation of energy-harvesting modules embedded within a flexible pavement structure. J. Transp. Eng. Part B Pavements.

[36] Huang Y., Wang L., Hou Y., Zhang W., Zhang Y. (2018). A prototype IOT based wireless sensor network for traffic information monitoring. Int. J. Pavement Res. Technol..

[37] Zhao H., Qin L., Ling J. (2018). Synergistic performance of piezoelectric transducers and asphalt pavement. Int. J. Pavement Res. Technol..

[38] Shi B., Shen S., Liu L., Wang X. (2021). Estimation of vehicle speed from pavement stress responses using wireless sensors. J. Transp. Eng. Part B Pavements.

[39] Mabrouk G., Elbagalati O., Dessouky S., Fuentes L., Walubita L. (2022). 3D-finite element pavement structural model for using with traffic speed deflectometers. Int. J. Pavement Eng..

[40] Yan G., Wang L., Ye Z., Wang W. (2020). Effects of crack damage on acceleration response of asphalt pavement via numerical analysis. J. Mater. Civ. Eng..

[41] Thulasibai A., Velayudhan S. (2022). Numerical modeling and optimization of the geometric properties influencing the deflection behavior of interlocking concrete block pavement. J. Transp. Eng. Part B Pavements.

[42] Thulasibai A., Velayudhan S., Pathath M., Lekshmipathy J., Visvanathan A. (2021). Experimental and numerical evaluation of the parameters influencing the shear-stress behavior of interlocking paver blocks-bedding sand interface using large-scale direct shear test. J. Mater. Civ. Eng..

[43] Matini N., Qiao Y., Sias J. (2022). Development of Time-Depth-Damage Functions for Flooded Flexible Pavements. J. Transp. Eng. Part B Pavements.

[44] Jamshidi A., Kurumisawa K., White G., Nishizawa T., Igarashi T., Nawa T., Mao J. (2019). State-of-the-art of interlocking concrete block pavement technology in Japan as a post-modern pavement. Constr. Build. Mater..

[45] Sun L., Wang G., Zhang H., Liu L. (2018). Initiation and propagation of top-down cracking in asphalt pavement. Appl. Sci..

[46] Lu G., Liu P., Törzs T., Wang D., Osser M., Grabe J. (2020). Numerical analysis for the influence of saturation on the base course of permeable pavement with a novel polyurethane binder. Constr. Build. Mater..

[47] Lu G., Wang H., Zhang Y., Liu P., Wang D., Oeser M., Grabe J. (2022). The hydro-mechanical interaction in novel polyurethane-bound pervious pavement by considering the saturation states in unbound granular base course. Int. J. Pavement Eng..

[48] Caro S., Manrique-Sanchez L., Kim Y. (2021). Computational evaluation of long-term raveling susceptibility of Permeable Friction Courses (PFC). Constr. Build. Mater..

[49] Walubita L., Ling M., Pianeta L., Fuentes L., Komba J., Mabrouk G. (2022). Correlating the asphalt-binder MSCR test results to the HMA HWTT and field rutting performance. J. Transp. Eng. Part B Pavements.

[50] Walubita L., Fuentes L., Prakoso A., Pianeta L., Komba J., Naik B. (2020). Correlating the HWTT laboratory test data to field rutting performance of in-service highway sections. Constr. Build. Mater..

[51] Zhang W., Chen X., Shen S., Mohammad L., Cui B., Wu S., Khan A. (2021). Investigation of field rut depth of asphalt pavements using hamburg wheel tracking test. J. Transp. Eng. Part B Pavements.

[52] Loizos A., Gkyrtis K., Plati C., Chabot A., Hornych P., Harvey J., Loria-Salazar L. (2020). Modelling Asphalt Pavement Responses Based on Field and Laboratory Data. Accelerated Pavement Testing to Transport Infrastructure Innovation. Lecture Notes in Civil Engineering.

[53] Islam M., Vallejo M., Tarefder R. (2017). Crack propagation in hot mix asphalt overlay using extended finite-element model. J. Mater. Civ. Eng..

[54] Sun Y., Zhang Z., Gong H., Zhou C., Chen J., Huang B. (2022). 3D multiscale modeling of asphalt pavement responses under coupled temperature–stress fields. J. Eng. Mech..

[55] Wang Y., Wang L., Maupin G. (2008). An inverse approach for evaluating the properties of asphalt concrete using the APA test. Road Mater. Pavement Des..

[56] Huang Y., Wang L., Xiong H. (2017). Evaluation of pavement response and performance under different scales of APT facilities. Road Mater. Pavement Des..

[57] Khan Z., Tarefder R. (2019). A procedure to convert field sensor data for finite element model inputs and its validation. Constr. Build. Mater..

[58] Ding Y., Wang H., Qian J., Zhou H. (2021). Evaluation of tire rolling resistance from tire-deformable pavement interaction modeling. J. Transp. Eng. Part B Pavements.

[59] Bai T., Cheng Z., Hu X., Fuentes L., Walubita L. (2021). Viscoelastic modelling of an asphalt pavement based on actual tire-pavement contact pressure. Road Mater. Pavement Des..

[60] Stache J., Robinson W. (2022). Effects of nonuniform tire contact pressures on near-surface pavement response. J. Transp. Eng. Part B Pavements.

[61] Zheng B., Chen J., Zhao R., Tang J., Tian R., Zhu S., Huang X. (2022). Analysis of contact behaviour on patterned tire-asphalt pavement with 3-D FEM contact model. Int. J. Pavement Eng..

[62] Wang T., Dong Z., Xu K., Ullah S., Wang D., Li Y. (2022). Numerical simulation of mechanical response analysis of asphalt pavement under dynamic loads with non-uniform tire-pavement contact stresses. Constr. Build. Mater..

[63] Liu X., Al-Qadi I. (2022). Three-dimensional tire-pavement contact stresses prediction by deep learning approach. Int. J. Pavement Eng..

[64] Behroozinia P., Khaleghian S., Taheri S., Mirzaeifar R. (2020). An investigation towards intelligent tyres using finite element analysis. Int. J. Pavement Eng..

[65] Said I., Hernandez J., Kang S., Al-Qadi I. (2020). Structural and environmental impact of new-generation wide-base tires in New Brunswick, Canada. Road Mater. Pavement Des..

[66] Peng J., Chu L., Fwa T. (2021). Determination of safe vehicle speeds on wet horizontal pavement curves. Road Mater. Pavement Des..

[67] Ding Y., Wang H. (2020). Computational investigation of hydroplaning risk of wide-base truck tyres on roadway. Int. J. Pavement Eng..

[68] Zhu X., Yang M., Bai S., Zhao H. (2022). A 3D virtual prototype-finite element co-simulation of aircraft hydroplaning on a wet rough runway. Int. J. Pavement Eng..

[69] Gerthoffert J., Cerezo V., Thiery M., Bouteldja M., Do M. (2020). A brush-based approach for modelling runway friction assessment device. Int. J. Pavement Eng..

[70] Schulz H., Curry J., Simões A. (2021). Water films and hydroplaning on highways: Hydrodynamic aspects. J. Transp. Eng. Part B Pavements.

[71] Luo J., Liu J., Wang Y. (2015). Validation test on pavement water film depth prediction model. China J. Highw. Transp..

[72] Luo W., Li L., Wang K., Wei C. (2020). Surface drainage evaluation of asphalt pavement using a new analytical water film depth model. Road Mater. Pavement Des..

[73] Luo W., Li L. (2021). Estimation of water film depth for rutting pavement using IMU and 3D laser imaging data. Int. J. Pavement Eng..

[74] Geng Y., Chen X., Chen Y., Ma Y., Huang X. (2019). Runoff characteristics for straightline segment asphalt pavement based on two-dimensional shallow water equations. J. Traffic Transp. Eng..

[75] Poshtmesari A., Nejad F. (2022). Analyzing moisture susceptibility of hot-mix asphalt based on tensile strength ratio, coating ratio, and thermodynamic parameters. J. Mater. Civ. Eng..

[76] Zhao Y., Jiang J., Zhou L., Ni F. (2022). Improving the calculation accuracy of FEM for asphalt mixtures in simulation of SCB test considering the mesostructure characteristics. Int. J. Pavement Eng..

[77] Zhao Y., Jiang L., Jiang J., Ni F. (2020). Accuracy improvement for two-dimensional finite-element modeling while considering asphalt mixture meso-structure characteristics in indirect tensile test simulation. J. Mater. Civ. Eng..

[78] Song W., Deng Z., Wu H., Zhan Y. (2022). Extended finite element modeling of hot mix asphalt based on the semi-circular bending test. Constr. Build. Mater..

[79] Chang M., Liu Z., Zhang J., Zhang Y., Yang B. (2022). Distribution characteristics of force chains in asphalt mixtures based on indirect tensile pattern. China J. Highw. Transp..

[80] Liu P., Lu G., Yang X., Jin C., Leischner S., Oeser M. (2021). Influence of different fillers on mechanical properties of porous asphalt mixtures using microstructural finite-element analysis. J. Transp. Eng. Part B Pavements.

[81] Bai X., Wang L. (2023). Study on mesoscopic model of low-temperature cracking of steel slag asphalt mixture based on random aggregate. Constr. Build. Mater..

[82] Nian T., Ge J., Li P., Wang M., Mao Y. (2021). Improved discrete element numerical simulation and experiment on low-temperature anti-cracking performance of asphalt mixture based on PFC2D. Constr. Build. Mater..

[83] Teng G., Zheng C., Chen X., Lan X., Zhu Y., Shan C. (2021). Numerical fracture investigation of single-edge notched asphalt concrete beam based on random heterogeneous FEM model. Constr. Build. Mater..

[84] Juliana M., Nilthson N., Carlos M., Celso R. (2020). Modeling of asphalt concrete fracture tests with the discrete-element method. J. Mater. Civ. Eng..

[85] Cezary S., Łukasz S., Marcin S., Jarosław G. (2022). The use of a two-phase Monte Carlo material model to reflect the dispersion of asphalt concrete fracture parameters. Theor. Appl. Fract. Mech..

[86] Sanfilippo D., Garcia-Hernández A., Alexiadis A., Ghiassi B. (2022). Effect of freeze–thaw cycles on the void topologies and mechanical properties of asphalt. Constr. Build. Mater..

[87] Erarslan N. (2023). Investigation of the tensile-shear failure of asphalt concrete base (ACB) construction materials using a non-linear cohesive crack model and critical crack threshold analysis. Constr. Build. Mater..

[88] Quezada J., Chazallon C. (2022). Discrete element modelling of hot mix asphalt complex modulus using realistic aggregate shapes. Road Mater. Pavement Des..

[89] Behnia B., Buttlar W., Reis H. (2018). Evaluation of low-temperature cracking performance of asphalt pavements using acoustic emission: A review. Appl. Sci..

[90] Sudarsanan N., Kim Y. (2022). A critical review of the fatigue life prediction of asphalt mixtures and pavements. J. Traffic Transp. Eng. (Eng. Ed.).

[91] Benaboud S., Takarli M., Pouteau B., Allou F., Dubois F., Hornych P., Nguyen M. (2021). Fatigue damage monitoring and analysis of aged asphalt concrete using acoustic emission technique. Road Mater. Pavement Des..

[92] Syrine C., Mondher N., Daniel P., Lotfi J. (2022). Numerical investigation to predict fatigue damage response in high-modulus asphalt mixture: A coupled damage-visco-elastoplastic approach. Int. J. Pavement Eng..

[93] Bertoldo C., Gorski R., Gonçalves R. (2020). Evaluation of elastic anisotropy of concrete using ultrasound wave propagation. J. Mater. Civ. Eng..

[94] Taghipoor M., Tahami A., Forsat M. (2020). Numerical and laboratory investigation of fatigue prediction models of asphalt containing glass wastes. Int. Fatigue.

[95] Papagiannakis A., Zelelew H., Mahmoud E. (2018). Simulation of asphalt concrete plastic deformation behavior. J. Mater. Civ. Eng..

[96] Peng Y., Gao H., Lu X., Sun L. (2020). Micromechanical discrete element modeling of asphalt mixture shear fatigue performance. J. Mater. Civ. Eng..

[97] Ge H., Quezada J., Houerou V., Chazallon C. (2021). Three-dimensional simulation of asphalt mixture incorporating aggregate size and morphology distribution based on contact dynamics method. Constr. Build. Mater..

[98] Yuan G., Li X., Hao P., Li D., Pan J., Li A. (2020). Application of flat-joint contact model for uniaxial compression simulation of large stone porous asphalt mixes. Constr. Build. Mater..

[99] Ji J., Wang Z., Yao H., Wang D., Zhang R., Diab A., Dai Q. (2021). A numerical study on rutting behaviour of direct coal liquefaction residue modified asphalt mixture. Road Mater. Pavement Des..

[100] Sadeghnejad M., Arabani M., Taghipoor M. (2018). Predicting the impact of temperature and stress on the glasphalt mixtures’ rutting behavior. Int. J. Pavement Res. Technol..

[101] Behera R., Das N., Nanthagopalan P. (2022). Development of simple and structured model for packing-density assessment of gap-graded coarse aggregates in concrete. J. Mater. Civ. Eng..

[102] Thilakarathna P., Baduge S., Mendis P., Chandrathilaka E., Vimonsatit V., Lee H. (2021). Aggregate geometry generation method using a structured light 3D scanner, spherical harmonics-based geometry reconstruction, and placing algorithms for mesoscale modeling of concrete. J. Mater. Civ. Eng..

[103] Kusumawardani D., Yiik W. (2020). Evaluation of aggregate gradation on aggregate packing in porous asphalt mixture (PAM) by 3D numerical modelling and laboratory measurements. Constr. Build. Mater..

[104] Pouranian M., Haddock J. (2021). A new framework for understanding aggregate structure in asphalt mixtures. Int. J. Pavement Eng..

[105] Jin C., Zou F., Yang X., Liu K. (2021). 3-D virtual design and microstructural modeling of asphalt mixture based on a digital aggregate library. Comput. Struct..

[106] Manrique-Sanchez L., Caro S., Estrada N., Castillo D., Alvarez A. (2022). Random generation of 2D PFC microstructures through DEM gravimetric methods. Road Mater. Pavement Des..

[107] Li Y., Wang L. (2020). Computer-aided procedure for analysis of effect of gradation and compaction temperature in asphalt mix design by using DEM. J. Transp. Eng. Part B Pavements.

[108] Ren J., Xu Y., Huang J., Wang Y., Jia Z. (2021). Gradation optimization and strength mechanism of aggregate structure considering macroscopic and mesoscopic aggregate mechanical behaviour in porous asphalt mixture. Constr. Build. Mater..

[109] Liu Y., Qian Z., Yang Y., Huang Q., Zhang X. (2022). Effect of curing reaction behaviors of warm mix epoxy asphalt concrete on its field compaction characteristics using discrete-element method. J. Mater. Civ. Eng..

[110] Chang J., Li J., Hu H., Qian J., Yu M. (2023). Numerical investigation of aggregate segregation of Superpave gyratory compaction and its influence on mechanical properties of asphalt mixtures. J. Mater. Civ. Eng..

[111] Elio Z., Fakhari Tehrani F., Beghin A., Petit C., Absi J., Millien A., Reynaud P. (2021). Experimental and numerical investigation on the rheological behaviour of bituminous composites via DSR testing. Road Mater. Pavement Des..

[112] Giancontieri G., Hargreaves D., Presti D. (2020). Are we correctly measuring the rotational viscosity of heterogeneous bituminous binders?. Road Mater. Pavement Des..

[113] Ye Z., Ren W., Yang H., Miao Y., Sun F., Wang L. (2021). An improved asphalt penetration test method. Materials.

[114] Wu J., Wang L., Hou Y., Qian Z., Meng L., Zhao Q. (2018). Simulation on the micro-deval test for the aggregate wear properties measurement. Constr. Build. Mater..

[115] Tan Z., Leng Z., Jiang J., Cao P., Jelagin D., Li G., Sreeram A. (2022). Numerical study of the aggregate contact effect on the complex modulus of asphalt concrete. Mater. Des..

[116] Cheng P., Yi J., Guo S., Pei Z., Feng D. (2022). Influence of fiber dispersion and distribution on flexural tensile properties of asphalt mixture Based on finite element simulation. Constr. Build. Mater..

[117] Polaczyk P., Ma Y., Jarrar Z., Jiang X., Xiao R., Huang B. (2023). Quantification of asphalt mixture interlocking utilizing 2D and 3D image processing. J. Mater. Civ. Eng..

[118] Hajikarimi P., Tehrani F., Nejad F., Absi J., Khodaii A., Rahi M., Petit C. (2019). Mechanical behavior of polymer-modified bituminous mastics. II: Numerical approach. J. Mater. Civ. Eng..

[119] Najmeddine A., Shakiba M. (2020). Impact of void morphology on the mechanical response of time-dependent heterogeneous media: A numerical investigation. J. Mater. Civ. Eng..

[120] Zhao G., Wang Q., Yan Z. (2021). Research on asphalt mixture bending test and micromechanical evolution based on 2D discrete-element method. J. Mater. Civ. Eng..

[121] Zhang K., Shen S., Lim J., Muhunthan B. (2019). Development of dynamic modulus-based mixture blending chart for asphalt mixtures with reclaimed asphalt pavement. J. Mater. Civ. Eng..

[122] Liu P., Xu H., Wang D., Wang C., Schulze C., Oeser M. (2018). Comparison of mechanical responses of asphalt mixtures manufactured by different compaction methods. Constr. Build. Mater..

[123] Tielmann M., Hill T. (2018). Air void analyses on asphalt specimens using plane section preparation and image analysis. J. Mater. Civ. Eng..

[124] Sahdeo S., Ransinchung G., Singh A. (2021). Microstructural and pore skeleton characteristics of pervious concrete containing RAP aggregates using X-ray microcomputed tomography and scanning electron microscope. J. Transp. Eng. Part B Pavements.

[125] Chandrappa A., Biligiri K. (2018). Pore structure characterization of pervious concrete using X-ray microcomputed tomography. J. Mater. Civ. Eng..

[126] Alexis E., Souza T., Aragão F., Braz D., Pereira A., Nogueira L. (2022). Determination of the air void content of asphalt concrete mixtures using artificial intelligence techniques to segment micro-CT images. Int. J. Pavement Eng..

[127] Yassine E., Fakhari F., Absi J., Courreges F., El M., Fatima A., Petit C. (2020). Modelling of asphalt mixes based on X-ray computed tomography and random heterogeneous generation. Int. J. Pavement Eng..

[128] Shakiba M., Darabi M., Rashid K., You T., Little D., Masad E. (2015). Three-dimensional microstructural modelling of coupled moisture–mechanical response of asphalt concrete. Int. J. Pavement Eng..

[129] Chen J., Wang J., Wang H., Xie P., Guo L. (2020). Analysis of pore characteristics and flow pattern of open-graded asphalt mixture in different directions. J. Mater. Civ. Eng..

[130] Meng A., Tan Y., Xing C., Lv H., Xiao S. (2020). Investigation on preferential path of fluid flow by using topological network model of permeable asphalt mixture. Constr. Build. Mater..

[131] Ghauch Z., Ozer H., Al-Qadi I. (2015). Micromechanical finite element modeling of moisture damage in bituminous composite materials. Constr. Build. Mater..

[132] Lv Q., Huang W., Bahia H., Tang N., Zhu T. (2018). Three-stage damage evolution of asphalt mixture in the wet Hamburg wheel tracking device test using X-ray computed tomography. J. Mater. Civ. Eng..

[133] Lövqvist L., Balieu R., Kringos N. (2021). A micromechanical model of freeze-thaw damage in asphalt mixtures. Int. J. Pavement Eng..

[134] You T., Abu Al-Rub R., Darabi M., Masad E., Little D. (2012). Three-dimensional microstructural modeling of asphalt concrete using a unified viscoelastic–viscoplastic–viscodamage mode. Constr. Build. Mater..

[135] Zadshir M., Hosseinnezhad S., Fini E. (2019). Deagglomeration of oxidized asphaltenes as a measure of true rejuvenation for severely aged asphalt binder. Constr. Build. Mater..

[136] Du Z., Zhu X., Zhang Y. (2021). Diffusive dynamics and structural organization of moisture in asphaltic materials based on molecular dynamics simulation. J. Mater. Civ. Eng..

[137] Qu X., Wang D., Hou Y., Oeser M., Wang L. (2018). Influence of paraffin on the microproperties of asphalt binder using MD simulation. J. Mater. Civ. Eng..

[138] Qu X., Wang D., Hou Y., Liu Q., Oeser M., Wang L. (2019). Investigation on self-healing behavior of asphalt binder using a six-fraction molecular model. J. Mater. Civ. Eng..

[139] Peng C., Lu L., You Z., Xu F., Zhou L., Ma H., Hu X., Liu Y. (2022). Influence of waste polyethylene on the performances of asphalt before and after oxidative aging based on the molecular dynamics simulation. J. Mater. Civ. Eng..

[140] Cao X., Deng M., Ding Y., Tang B., Yang X., Shan B., Su Y. (2021). Effect of photocatalysts modification on asphalt: Investigations by experiments and theoretical calculation. J. Mater. Civ. Eng..

[141] Zhu X., Du Z., Ling H., Chen L., Wang Y. (2020). Effect of filler on thermodynamic and mechanical behaviour of asphalt mastic: A MD simulation study. Int. J. Pavement Eng..

[142] Khan Z., Faisal H., Tarefder R. (2018). Evaluation of nanomechanical properties of nonaggregate phase of asphalt concrete using finite-element method. J. Mater. Civ. Eng..

[143] Apostolidis P., Scarpas A. (2020). Numerical study of sorption of asphalt binders on minerals. Constr. Build. Mater..

[144] Yaphary Y., Leng Z., Wang H., Ren S., Lu G. (2022). Characterization of nanoscale cracking at the interface between virgin and aged asphalt binders based on molecular dynamics simulations. Constr. Build. Mater..

[145] Andres C., Caro S., Lleras M., Rojas Y. (2019). Impact of the chemical composition of aggregates on the adhesion quality and durability of asphalt-aggregate systems. Constr. Build. Mater..

[146] Zhai M., Li J., Wang R., Yue J., Wang X. (2023). Revealing mechanisms of aging and moisture on thermodynamic properties and failure patterns of asphalt-aggregate interface from the molecular scale. J. Mater. Civ. Eng..

[147] Wang W., Luo R., Yang H., Wang L. (2022). Analysis of water erosion on asphalt binder using multi-scale experimental methods. Int. J. Pavement Res. Technol..

[148] Du Z., Zhu X., Li F., Zhou S., Dai Z. (2021). Failure of the asphalt-aggregate interface under tensile stress: Insight from molecular dynamics. J. Mater. Civ. Eng..

[149] Du Z., Zhu X. (2022). Surface-roughness-induced control of the interfacial failure mode and bonding strength: Atomistic case study in an asphalt-aggregate system. J. Mater. Civ. Eng..

[150] Zhang L., Long N., Liu Y., Wang L. (2022). Cross-scale study on the influence of moisture-temperature coupling conditions on adhesive properties of rubberized asphalt and steel slag. Constr. Build. Mater..

[151] Wang L., Zhang L., Liu Y. (2022). Molecular dynamics study on the effect of mineral composition on the interface interaction between rubberized asphalt and aggregate. J. Mater. Civ. Eng..

[152] AbuQtaish L., Nazzal M., Kaya S., Kim S., Abbas A., Abu H. (2018). AFM-based approach to study blending between RAP and virgin asphalt binders. J. Mater. Civ. Eng..

[153] Nazzal M., Qtaish L., Al-Hosainat A., Talha S., Kaya S., Abbas A. (2021). Evaluation of moisture damage in asphalt mixtures at macro- and nanoscales. J. Mater. Civ. Eng..

[154] Tarefder R., Zaman A. (2010). Nanoscale evaluation of moisture damage in polymer modified asphalts. J. Mater. Civ. Eng..

[155] Macedo T., Badilla-Vargas G., Osmari P. (2020). An experimental testing and analysis procedure to determine linear viscoelastic properties of asphalt binder microstructural components. Constr. Build. Mater..

[156] Shan L., Zhang E., Liu S., Xu H., Tan Y. (2020). Analysis of microscopic damage mechanism of asphalt binder through atomic force microscopy (AFM). China J. Highw. Transp..

[157] Du C., Liu P., Ganchev K., Lu G., Oeser M. (2021). Influence of microstructure evolution of bitumen on its micromechanical property by finite element simulation. Constr. Build. Mater..

